# Implementing the H&P 360 in Three Medical Institutions: Usability Study

**DOI:** 10.2196/66221

**Published:** 2025-06-05

**Authors:** Rupinder Hayer, Joyce Tang, Julia Bisschops, Gregory W Schneider, Kate Kirley, Tamkeen Khan, Erin Rieger, Eric Walford, Irsk Anderson, Valerie Press, Brent Williams

**Affiliations:** 1 American Medical Association Improving Health Outcomes Chicago, IL United States; 2 Section of Hospital Medicine University of Chicago Pritzker School of Medicine Chicago, IL United States; 3 Department of Humanities, Health and Society Herbert Wertheim College of Medicine Florida International University. Miami, FL United States; 4 Florida International University Miami, FL United States; 5 Columbia University Medical Center New York, NY United States; 6 University of Michigan Ann Arbor, MI United States

**Keywords:** history and physical, medical education, social drivers, social determinants of health

## Abstract

**Background:**

The traditional history and physical (H&P) provides the basis for physicians’ data gathering, problem formulation, and care planning, yet it can miss relevant behavioral or social risk factors. The American Medical Association’s “H&P 360,” a modified H&P, has been shown to foster information gathering and patient rapport in inpatient settings and objective structured clinical examinations. It prompts students to explore 7 domains, as appropriate to the clinical context: biomedical problems, psychosocial problems, patients’ priorities and goals, behavioral history, relationships, living environment and resources, and functional status.

**Objective:**

This study aims to examine the perceived usability of the H&P 360 outside standardized patient settings.

**Methods:**

The H&P 360 was implemented in various clinical settings across 3 institutions. Of the 207 student participants, 18 were preclerkship, 126 were clerkship, and 63 were postclerkship; 3-8 months after implementation, we administered a student survey consisting of 14 Likert-type items (1=strongly disagree to 5=strongly agree) and 3 free-text response items to assess usability.

**Results:**

Of the 207 students, 61 responded to the survey (response rate was 29.5%). Among all students, mean ratings on the 3 usability survey items ranged from 4.03 to 4.24. The 5 items assessing the impact on patient care had mean ratings ranging from 3.88 to 4.24. The mean ratings for the 2 student learning items were 4.10 and 4.16. Students’ open-ended comments were generally positive, expressing a perceived value in obtaining a more complete contextual picture of patients’ conditions and supporting the usability of the H&P 360. Survey response patterns varied across institutions and learner levels.

**Conclusions:**

Our findings suggest that using the H&P 360 may enhance information gathering critical for chronic disease management, particularly regarding social drivers of health. As a potential new standard, the H&P 360 may have clinical usability for identifying and addressing health inequities. Future work should assess its effects on patient care and outcomes.

## Introduction

The traditional history and physical (H&P) structure is central to the patient-physician interaction and remains a foundational element of medical education. Through medical history, physicians elicit 60%-80% of the information relevant to diagnosis and treatment [1]. Medical students are typically required to master the skill of gathering, synthesizing, and documenting patient information early in their training. The traditional H&P, used in most medical education settings and routine clinical practice, is primarily structured to diagnose acute medical conditions and has not evolved for generations—despite the growing prevalence of chronic diseases and the increasing influence of social and behavioral drivers of health [2,3].

The social determinants or social drivers of health (SDOH) heavily influence the health of patients and populations [4]. The World Health Organization (WHO), in its conceptual framework for action on SDOH, defines “social determinants of health” as the full set of social conditions in which people live and work [4,5]. We will use the phrases social determinants and social drivers interchangeably. We will refer to social risk factors, meanwhile, as individual-level adverse SDOH, such as housing instability or low education level, and social needs as social factors that take into account people’s individual preferences and priorities in identifying and guiding social interventions [6,7].

Health systems and providers are increasingly exploring ways to better integrate health care delivery with reforms aimed at addressing the SDOH, identifying patients’ social risk factors, and meeting patients’ social needs [8,9]. At the upstream level, laws, policies, and regulations can be used to create community conditions that foster health. At the midstream level, providers and health systems can include screening questions to identify social risk factors and offer services that connect patients to resources to meet their social needs. At the downstream level, clinicians can tailor medical interventions to acknowledge individual social conditions [6,8,9]. To significantly improve the health of all, it is critical to emphasize addressing the broader SDOH inequities—those created and sustained by structural racism and the marginalization of specific groups, including women, Black, Hispanic/Latino, LGBTQ+ (lesbian, gay, bisexual, transgender, queer or questioning, and other diverse sexual orientations and gender identities) individuals, people living with disabilities, and other populations [4,5,10-12].

In addition to increased attention to the SDOH, both globally and particularly in the United States, chronic diseases are the largest contributors to disease burden, accounting for 90% of health care costs in the United States [13]. Health systems are increasingly addressing upstream factors, such as behavioral health, and social and environmental circumstances, to prevent and manage chronic diseases and their consequences [14,15]. Against this multidimensional backdrop, individual clinicians, residents, and medical students typically rely on the H&P examination as their principal method of information gathering. However, when using the traditional H&P to frame and organize this process, learners at all levels are not prompted to collect relevant biopsychosocial data, including social needs and health behaviors, which are key to preventing and managing chronic diseases. Recent research has shown that inaccurate and incomplete patient histories are among the leading causes of diagnostic errors [16].

The H&P 360, a modified version of the traditional H&P, was developed by the American Medical Association’s (AMA) Chronic Disease Prevention and Management interest group in May 2017, building on earlier work by medical educators at the University of Michigan (UM) [12,17]. This new approach was designed to more explicitly acknowledge the SDOH, the prevalence of chronic diseases, and the importance of patients’ preferences and priorities in clinical decision-making. It intentionally incorporates the WHO conceptual framework for addressing the SDOH at the micro-level of individual interaction [4,5], the Centers for Disease Control and Prevention (CDC) framework for addressing chronic diseases at the health system and community-clinic levels [12,18], and contemporary models of shared decision-making [1,19].

The H&P 360 is grounded in the idea that the central, standardized written template in medical education (ie, the traditional H&P) plays a significant role in both enhancing and constraining information-seeking related to medical decision-making. This understanding of information gathering aligns with Structuration Theory and Cognitive Load Theory. Structuration Theory posits that social practice both shapes and is shaped by the structures, such as learning templates, within which it occurs [20]. Cognitive Load Theory, meanwhile, asserts that cognitive capacity is limited and that learning is enhanced when key information is presented in manageable blocks, such as the 7 domains [21].

At a deeper level, the H&P may play an important role in shaping physicians’ professional identity and role expectations, as suggested by Social Learning Theory [22,23], which posits that individuals’ agency and role identities are critically influenced by the social and institutional contexts in which they develop. The H&P 360 prompts students to collect relevant biopsychosocial information, particularly social risk factors and needs, using a systematic yet flexible framework. While it retains the basic structure of the traditional H&P for eliciting biomedical information, the H&P 360 also includes general prompts for 6 additional domains: patients’ priorities and goals, psychosocial problems, behavioral history, relationships, living environment and resources, and functional status. The 6 nonbiomedical domains were identified through a literature review as those consistently represented in comprehensive clinical assessment settings, including geriatrics, and care for homeless and chronically mentally ill persons, as well as in the categories of the Diagnostic and Statistical Manual of Mental Disorders (4th edition) [24], and have been applied in numerous clinical and teaching settings since 2010 [17]. See Multimedia Appendix 1 for the H&P 360 template.

When using the H&P 360, students are encouraged to ask a few questions from each of the domains as part of the standard history. These additional questions help students gain a more comprehensive understanding of a patient’s biopsychosocial condition and support the development of an appropriate treatment and management plan. In follow-up encounters, continued exploration of the 7 H&P 360 domains can foster a deeper understanding of the patient, informing chronic disease management. A previous randomized trial conducted at 4 medical schools found that medical students using the H&P 360 in a standardized patient setting collected significantly more biopsychosocial information compared with students using the traditional H&P [3]. Another study found that students who applied the H&P 360 using templated notes in the electronic health record reported improved elicitation of patient goals and perspectives, as well as identification of contextual factors and patient needs critical to preventing rehospitalization [25]. In addition to enhanced data gathering, the H&P 360 has been shown to encourage multidisciplinary team care planning [17] and to improve patient rapport (unpublished data).

The goal of this study was to examine the perceived usability of the H&P 360 by both faculty and students across a variety of clinical settings and learner levels in routine clinical teaching contexts. To assess usability in different clinical teaching environments, the AMA launched a grant program for institutions willing to implement the H&P 360 in student clinical encounters and administer a standardized postintervention survey to faculty and students across sites. We hypothesized that students and faculty across sites would appreciate the usability of the approach, but that barriers to engagement would vary by site and learner level.

## Methods

### Site Selection

The AMA offered funding for projects to implement the H&P 360 within clinical settings at academic institutions. Priority was given to projects aimed at developing additional supporting materials and gathering student and faculty feedback during the implementation phase. Following a call for proposals, 4 academic institutions received grants from the AMA to implement the H&P 360 across a diverse range of clinical settings and undergraduate medical education learner levels. The grant period began in January 2020 and ended in June 2021. Because of the pandemic, only 3 of the institutions were able to implement their grants. These institutions were the UM School of Medicine, the University of Chicago Pritzker School of Medicine (UC), and the Herbert Wertheim College of Medicine at Florida International University (FIU). The fourth institution was unable to implement its grant project but still incorporated the H&P 360 with its students.

The 3 grant-funded institutions implemented the H&P 360 across a variety of clinical settings and learner levels, described in detail in Table 1. Clinical settings included inpatient, outpatient, virtual, community-based clinics, and longitudinal outpatient clinics. Learner levels ranged from preclerkship to clerkship and postclerkship. The approaches each school used to introduce students and faculty to the H&P 360 are also detailed in Table 1. At all 3 sites, students were introduced to the H&P 360 through a nonstandardized 1- to 2-hour seminar. One site (UC) implemented standardized note templates within the electronic health record to facilitate documentation of the H&P 360. Faculty orientation to the H&P 360 varied across sites, ranging from emailed communications with attached introductory materials and a teaching guide (UM and UC) to virtual orientation sessions over Zoom (Zoom Communications, Inc) and some in-person sessions (FIU and UC).

**Table 1:** Learner level, clinical setting, and teaching context by institution.

Approaches to introducing H&P^a^ 360	Learner level			Students, n	Setting	Duration of use	How the H&P 360 was introduced to students and topics covered	How the H&P 360 was introduced to faculty and topics covered
	Preclerkship	Clerkship	Postclerkship					
The University of Chicago Pritzker School of Medicine	✓	N/A^b^	N/A	18	Preclerkship students were encouraged to utilize the H&P 360 in a longitudinal patient-partnered clinical experience (about 6 face-to-face and 2 virtual clinical sessions).	9 months	Preclerkship students attended a presentation on the format and components and received training materials including examples and the interview guide.	Faculty precepting preclerkship students attended a presentation or received an email.
The University of Chicago Pritzker School of Medicine	N/A	✓	N/A	8	Clerkship students were encouraged to utilize the H&P 360 during COVID-19 follow-up virtual visits.	1 month	Clerkship students attended a 1-hour virtual training session with background about the H&P 360 and details on using a COVID-19–specific note template. The supporting interview guide and pocket card were leveraged as needed.	Faculty attended 2 or more presentations on the H&P 360.
The University of Chicago Pritzker School of Medicine	N/A	N/A	✓	24	Postclerkship students were encouraged to utilize the H&P 360 in an internal medicine subinternship for 1 admission per call cycle.	1 month	Postclerkship students received an email from their course director with background about the H&P 360 and instructions to access H&P 360 templates. The supporting interview guide and pocket card were leveraged as needed.	Faculty supervising postclerkship students received an email.
University of Michigan School of Medicine	N/A	N/A	✓	39	All students utilized the H&P 360 in an outpatient setting. Of the 39 students, 8 used it in a community-based elective and 31 used it in a longitudinal clinic setting. Students were encouraged to apply the H&P 360 in every encounter.	Elective (1 month) and longitudinal clinics (9 months)	A 2-hour interactive in-person seminar with case examples. The supporting interview guide and pocket card were leveraged as needed.	Email introduction and follow-up, which included teaching tips, pocket cards, profiles, and cases.
Herbert Wertheim College of Medicine at Florida International University	N/A	✓	N/A	118	All students utilized the H&P 360 in a virtual and longitudinal, interprofessional, home-based service-learning program.	12 months/1 academic year as part of a longitudinal program.	Introduced during an interactive didactic session on chronic disease management; the self-directed video was also available for students.	One in-person faculty development session before COVID-19; a self-directed video for faculty; and a conference presentation by Dr. Brent Williams from the University of Michigan.

^a^H&P: history and physical.

^b^N/A: not applicable.

### Settings

At FIU, the investigators had to alter their initial implementation strategy due to the COVID-19 pandemic. The project team completed 1 in-person faculty orientation; subsequent sessions were delivered virtually as an online module tailored to both faculty and students. For students, the team relied solely on the online module, as the planned in-person session was canceled due to the pandemic.

At UC, initial implementation plans were also disrupted by the COVID-19 pandemic, which led to medical students being removed from traditional clinical settings. During this period, an innovative program was developed in which clerkship students conducted phone outreach to patients newly testing positive for COVID-19. Taking advantage of this novel opportunity, the H&P 360 was used to help structure these outreach calls. Because of the small number of students and faculty involved, an in-depth, interactive training program for both faculty and students was offered via Zoom meetings. In the later implementation of the H&P 360 for preclerkship students, a virtual orientation over Zoom was incorporated into their Clinical Skills course. Because of the large number of faculty serving as preceptors for this course, only new faculty preceptors—who were required to attend a mandatory orientation session—received virtual training on the H&P 360 framework. Preceptors who were not new to the program and for whom orientation attendance was not required received emailed communication about the H&P 360. Implementation for postclerkship students was further modified to email communication only. These students had rolling start times each month, and there was no formal orientation session during the clerkship to integrate separate training. The number of clinical faculty preceptors for postclerkship students was quite large, also with rolling start times every 2 weeks, making email communication regarding the H&P 360 the most feasible approach.

At UM, the H&P 360 was implemented during the postclerkship period in 2 settings: a 1-month clinical elective focused on underserved populations before the pandemic (8 students over 2 months), taught by 1 of the authors (BW), and longitudinal weekly clinics in primary care settings over 9 months during the pandemic (31 students). The longitudinal clinic rotation was chosen due to the faculty coordinator’s interest in implementing the H&P 360 and its suitability for continuity settings, where the domains can be explored with patients over time. For the longitudinal rotation, students received a 2-hour introduction to the H&P 360, including case examples. Precepting faculty were sent introductory materials, teaching tips, and written case examples via email both at the start and several months into the longitudinal clinics. Many longitudinal clinics transitioned to telemedicine visits during the pandemic. In both rotations, students were encouraged—but not required—to use the H&P 360, or portions of it, in every encounter.

### Survey Structure

Data collection consisted of a student survey on using the H&P 360 in undergraduate medical education settings. As the survey focused specifically on the use of the H&P 360, previously published surveys were not applicable. Theoretical frameworks guiding survey development included Bloom’s taxonomy of learning objectives [26], which emphasizes synthesis and application of knowledge rather than factual recall, and the Expectancy-Value Theory of Motivation [27], which posits that learner motivation is influenced by the perceived value of new information.

The survey consisted of an initial section asking for examples of a question relevant to each of the 5 domains from the H&P 360, followed by 14 Likert-type items (response scale: 1=strongly disagree to 5=strongly agree) and 3 open-ended questions. The Likert-type items were developed using a “blueprint” of 7 potential impact areas of the H&P 360, designed by the authors. Source items were either adapted from a 10-item version used in a previous study [17] or newly created. To minimize the response burden, the survey was limited to 15 items or fewer. Items were reviewed for sensibility by small groups of medical students and residents not involved in the study, resulting in minor modifications. The final instrument included 14 Likert-type items. Two items were modified or omitted at some sites and thus were not included in the analyses. The analyzed items are shown in Tables 2 and 3. The 7 areas from the “blueprint” and their corresponding item numbers were perceived usability of the H&P 360 (items 1, 2, and 3); impact on history-taking (item 4); perceived clinical value added (items 5 and 6); promotion of understanding patients’ goals (item 7); enhancement of patient-provider relationships (item 8); facilitation of care planning (item 9); and promotion of inclusion of other health professionals (item 10). Two additional items were included as global measures of educational and clinical value, respectively (items 11 and 12). By covering a broad range of topics, results from individual items could be used independently by educators to inform a wide spectrum of educational and research activities.

The 3 open-ended questions were designed to elicit specific feedback about the H&P 360: “Name two (or more) aspects of the H&P 360 you found helpful”; “Name two (or more) aspects of the H&P 360 you found challenging”; and “What changes would you recommend for the H&P 360?” A systematic review of the open-ended comments is not included in this paper. Instead, a subset of comments reflecting students’ perceived value and limitations of the H&P 360 is provided in Multimedia Appendix 2.

**Table 2:** Mean student survey scores (Likert scale 1-5)^a^ by school.

Student survey scores by school			All students (N=49) (N=61)^b^, mean (SD)		All FIU^c^ students (N=17), mean (SD)		All UM^d^ students (N=13), mean (SD)		All UC^e^ students (N=19) (N=31)^b^, mean (SD)
**Usability**									
	1. The H&P^f^ 360 was easy to use	4.12 (0.78)		4.18 (0.64)		4.15 (0.69)		4.05 (0.97)	
	2. Elements of the H&P 360 are potentially useful in all patient interactions	4.24 (0.90)		4.35 (0.61)		4.62 (0.51)		3.89 (1.20)	
	3. I plan to use the H&P 360 during other rotations^b^	4.03 (0.84)		3.59 (0.94)		4.31 (0.75)		4.16 (0.73)	
**Impact on patient care**									
	4. The H&P 360 changed some of the questions I ask patients during the encounter	4.08 (0.76)		3.59 (0.87)		4.46 (0.52)		4.26 (0.56)	
	5. The H&P 360 helped create a more comprehensive problem list	3.88 (0.95)		4.06 (0.90)		4.15 (0.69)		3.53 (1.07)	
	6. The H&P 360 added valuable information that I would not otherwise know about the patient^b^	4.18 (0.79)		3.82 (1.01)		4.46 (0.78)		4.26 (0.58)	
	7. The H&P 360 helped me better understand patients’ goals^b^	4.15 (0.68)		4.12 (0.78)		4.23 (0.44)		4.13 (0.72)	
	8. Using the H&P 360 facilitated a stronger provider-patient relationship^b^	4.24 (0.67)		4.12 (0.70)		4.08 (0.64)		4.35 (0.66)	
	9. I was able to develop management plans that incorporated information from the H&P 360	3.88 (0.83)		4.00 (0.87)		4.00 (0.41)		3.68 (1.00)	
**Overall impact on student learning**									
	10. The H&P 360 helped me learn to be a better clinician^b^	4.16 (0.64)		4.06 (0.66)		4.31 (0.63)		4.16 (0.64)	
	11. The H&P 360 helped improve the care I provided to my patients	4.10 (0.71)		4.06 (0.75)		4.31 (0.63)		4.00 (0.75)	

^a^1=strongly disagree to 5=strongly agree.

^b^These are items with a greater number of respondents because an abbreviated version of the survey was completed by preclinical students at UC.

^c^FIU: Florida International University.

^d^UM: University of Michigan.

^e^UC: University of Chicago.

^f^H&P: history and physical.

**Table 3:** Mean student survey scores (Likert scale 1-5)^a^ by clerkship status.

Mean student survey scores				Preclerkship students (N=15), mean (SD)		Clerkship students (N=25), mean (SD)		Postclerkship students (N=24), mean (SD)		
**Usability**										
		1. The H&P^b^ 360 was easy to use		N/A^c^		4.32 (0.63)		3.92 (0.88)		
		2. Elements of the H&P 360 are potentially useful in all patient interactions		N/A		4.40 (0.76)		4.08 (1.02)		
		3. I plan to use the H&P 360 during other rotations		4.33 (0.49)		3.84 (0.90)		4.08 (0.88)		
**Impact on patient care**										
		4. The H&P 360 changed some of the questions I ask patients during the encounter		N/A		3.92 (0.91)		4.25 (0.53)		
		5. The H&P 360 helped create a more comprehensive problem list		N/A		4.08 (0.86)		3.67 (1.21)		
		6. The H&P 360 added valuable information that I would not otherwise know about the patient		4.25 (0.45)		4.04 (0.93)		4.29 (0.75)		
		7. The H&P 360 helped me better understand patients’ goals		4.25 (0.45)		4.12 (0.83)		4.13 (0.61)		
		8. Using the H&P 360 facilitated a stronger provider-patient relationship		4.17 (0.58)		4.36 (0.70)		4.13 (0.68)		
		9. I was able to develop management plans that incorporated information from the H&P 360		N/A		4.08 (0.81)		3.67 (0.82)		
		10. The H&P 360 facilitated care planning that included other health professionals		3.75 (0.97)		4.08 (0.86)		4.00 (1.02)		
**Overall impact on student learning**										
	11. The H&P 360 helped me learn to be a better clinician		4.25 (0.45)		4.20 (0.65)		4.08 (0.12)			
	12. The H&P 360 helped improve the care I provided to my patients		N/A		4.20 (0.71)		4.00 (0.72)			

^a^1=strongly disagree to 5=strongly agree.

^b^H&P: history and physical.

^c^N/A: not applicable.

The survey was administered online by all 3 sites approximately 3-8 months after implementation. Six items that presumed experience in clinical care were not administered to preclinical students participating in this study; this omission applied only to a subset of students at 1 site. Data were aggregated across all sites to calculate mean scores and SDs for each survey item, allowing comparisons by institution and by clerkship status. Because of the small number of respondents in each subgroup, we were limited to analyzing descriptive statistics and were unable to conduct psychometric analyses or hypothesis testing to statistically compare subgroups. However, the descriptive analysis was still useful for aggregating data across multiple sites and generating hypotheses. Data analysis was conducted using STATA version 13.0 (StataCorp). See Multimedia Appendix 3 for the supporting CHERRIES (Checklist for Reporting Results of Internet E-Surveys) document.

### Ethics Considerations

The UM received exempt institutional review board status from the Institutional Review Boards of the UM Medical campus. FIU received exempt institutional review board status from The FIU Office of Research Integrity. The UC received exempt institutional review board status from the BSD/UCMC Institutional Review Boards at the UC. Lastly, the AMA confirmed that this study was not deemed to be research by the University of Illinois Chicago Institutional Review Board. All 4 institutions confirmed that all methods were carried out in accordance with relevant guidelines and regulations.

## Results

### Summary of the Survey Findings

The Likert-type survey items were organized by consensus among the authors into 3 sets to identify patterns and facilitate discussion: Usability (3 items); Impact on Patient Care (7 items); and Overall Impact on Student Learning (2 items). Results are presented for all student respondents by institutional site in Table 2 and by learner-level subgroups in Table 3. Of the 207 students, 61 (29.5%) responded to the survey. Institutional response rates were as follows: FIU, 17 out of 118 (14.4%) students; UM, 13 out of 39 (33.3%) students; and UC, 31 out of 50 (62.0%) students.

Among all students, mean ratings on the 3 survey items related to usability (ease of use, use in all encounters, and intention to use in other rotations) were high, with mean (SD) scores ranging from 4.03 (0.84) to 4.24 (0.90) (Table 2). Some students’ comments suggested that efficiently using the H&P 360 requires practice. One postclerkship student commented: “(The H&P 360)...is quite long so it was challenging to hit aspects of each domain while attempting to time manage. However, hitting 1-item from each domain, chosen on a case-by-case basis, seems quite doable.” Several students raised concerns about the awkwardness of asking some questions, particularly during virtual outreach calls to patients who had newly tested positive for COVID-19. One clerkship student commented: “It did not always feel natural to fit into the conversation with every patient. Some were not very open to conversation, which is understandable since we were strangers calling them out of the blue.”

Students also found that the H&P 360 positively affected patient care by expanding the range of information available for clinical decision-making and promoting stronger patient-clinician relationships. Mean ratings across the 5 related items ranged from 3.88 (SD 0.95) to 4.24 (SD 0.67; see Table 3 for details). Student feedback on clinical impact emphasized the benefits of the H&P 360 in building rapport. One clerkship student commented: “It helped me build rapport with my patient and have a better [understanding] of their life and how it affects their health.” Others mentioned that the H&P 360 helped build trust and identify high-risk situations. See Multimedia Appendix 2 for additional relevant student comments.

Students also found that the H&P 360 facilitated their learning and development as clinicians, with mean ratings of 4.10 (SD 0.71) and 4.16 (SD 0.64) for the items “the H&P 360 helped me...improve the care I provided to my patients” and “…be a better clinician,” respectively. One postclerkship student commented: “The H&P 360 was helpful in...[r]eturning the humanity to medicine: patients are people first—Helping to understand some of the barriers to health and disease prevention that might not otherwise be apparent.”

### Site-Specific Survey Findings

Some variation in student survey responses was observed across institutions (Table 2). For 2 items related to using the H&P 360 to develop problem lists and management plans, student ratings at UC were lower than those at UM or FIU. For 3 items—related to using the H&P 360 in other rotations and its role in changing some questions and adding valuable information—student ratings at FIU were lower compared with UC and UM.

### Survey Findings by Learner Levels

Across learner levels, some variation in student survey responses was noted for a minority of items (Table 3). For example, preclinical students gave relatively low ratings on the item related to facilitating care planning that included other health professionals compared with their responses on other items. While clerkship students valued the H&P 360 in all patient interactions and for facilitating stronger patient relationships, their ratings were relatively low for items related to the H&P 360 changing the questions they asked and their plans to use it in future rotations. Postclerkship students gave high ratings for 9 of the 12 questions. Lower ratings were observed for items related to ease of use, creating a more comprehensive problem list, and the ability to develop management plans incorporating information from the H&P 360. The phrasing of the item on time burden evolved over time and was therefore not administered consistently across or within institutions.

## Discussion

### Principal Findings

Previous work has documented the advantages of the H&P 360 over the traditional H&P during single inpatient encounters [17] and with standardized patients [3]. This study examined the use of the H&P 360 across a broad range of routine, longitudinal clinical teaching settings. Medical students at 3 institutions, spanning different levels of training and diverse ambulatory, inpatient, community, and virtual settings, found the H&P 360 useful and reported a positive impact on patient care and their own learning. The perceived benefits of the H&P 360 include helping students gather relevant information on patients’ goals and circumstances, as well as potential barriers and facilitators of health. It also enhances patient-provider relationships and encourages interprofessional care planning. Compared with the traditional H&P, student feedback suggests that the H&P 360 made them better clinicians. We can further speculate that by using the H&P 360, students develop a more complete picture of the patient—not just signs, symptoms, and diagnoses—but also the social and human narrative context that critically influences the presentation, management, and ultimately the outcomes of disease conditions. We suspect that gathering this more complete picture of patients’ lives is one factor contributing to students’ perception that the provider-patient relationship was enhanced by using the H&P 360. An important area for future investigation is the mechanism behind this enhanced relationship. Perhaps it is this more complete understanding of the patient, combined with improved patient rapport, that prompted the student comment that the H&P 360 “...return(ed) the humanity to medicine.”

### Implication of Findings

The observed variation across institutions and learner levels—though limited by small sample sizes and collinearity between institutions and learner levels—may offer insights into factors influencing medical students’ perceived value of the H&P 360. Here, we present 3 speculative observations based on these data to encourage future research and application of the H&P 360. First, the teaching setting for the FIU students included in this study was a community-based, longitudinal, interprofessional environment with an established strong emphasis on comprehensive assessment and interprofessional care. As such, FIU students may have been less likely to perceive that the H&P 360 changed questions asked or added valuable information beyond their prior practice. Additionally, during the study period, FIU students conducted visits virtually due to COVID-19. Second, UC provided a relatively unique perspective on implementation efforts by including preclinical students and applying the H&P 360 in telehealth settings. Although the very high perceived value of the H&P 360 among both preclinical students and clinical-year students in telehealth settings is striking and promising, further investigation in other settings and institutions is needed to contextualize these findings. Finally, variation among institutions may reflect differences in overall emphasis on SDOH; the role of faculty in promoting or minimizing the study findings and application of the H&P 360; or differences in its use across inpatient, outpatient, and virtual settings.

Synthesizing evidence on the H&P 360 from this and previous studies, along with input from faculty and students and our own clinical teaching experience, we suggest that incorporating the H&P 360 into routine clinical practice involves at least 4 dimensions of learning. The general patterns and variations observed across different learner levels and clinical settings in this study support and shed light on each of these dimensions.

First, learners and educators using the H&P 360 will need to *integrate domain-based thinking alongside checklist-based approaches in data gathering.* Currently, early medical students are taught to take a history by following a memorized list of specific questions. Over time, an implicit process develops, where clinicians tailor questions, diagnoses, and management plans based on patient-specific information [28]. The direction a clinician takes for follow-up inquiry is likely influenced by many factors, including training experiences, knowledge and clinical skills in managing a wide range of issues (eg, emotional well-being, food insecurity, or safe housing), and local practice norms. Consequently, this approach is likely to vary widely among clinicians. Some naturally explore psychosocial dimensions, while others remain more narrowly focused on biomedical factors. To reduce this variation and better address the role of psychological and social factors in patients’ health, the H&P 360 provides uniform, systematic prompts that help clinicians recognize social and psychological determinants of health. The H&P 360 represents a fundamental shift in learning to gather patient information by introducing 6 domains as general reference points alongside the traditional checklist focused on biomedical information.

For early learners, balancing domain-based thinking with a checklist approach can be disconcerting as they decide which specific content to include or exclude within the nonbiomedical domains. This challenge aligns with student feedback that the H&P 360 initially feels long and overwhelming when seen as a checklist of individual items, but becomes manageable and useful when viewed as a set of domain-based prompts that can be selectively explored—or revisited over multiple patient encounters. This is also consistent with findings that senior medical students using the H&P 360 identify and apply significantly more psychosocial information in their care planning than those using the traditional H&P [17]. Additionally, our finding that preclinical students found the H&P 360 added valuable information, helped them understand patients’ goals, and facilitated stronger patient-provider relationships suggests that early learners can successfully incorporate domain-based thinking into routine data gathering. The interview guide that accompanies the H&P 360 can be a valuable resource in this regard. It helps students decide which domain to focus on and also supports faculty in navigating these domains during classroom teaching. See Multimedia Appendix 4 for the H&P 360 interview guide. Further exploration of domain-based thinking among medical learners is warranted to identify the best ways to provide a data-gathering framework that is both accessible in the early stages and comprehensive in the later stages of learning. We are particularly interested in methods for—and the implications of—incorporating patients’ values, priorities, and goals into every clinical encounter [29,30].

Second, once familiar with domain-based thinking, medical students need to *develop skills in deciding which specific information within a domain is most relevant to a given patient encounter.* The finding that clerkship and postclerkship students from both inpatient and outpatient settings found the H&P 360 added valuable information and enhanced patient care suggests that students perceive tailoring domain-based questions to individual patients and clinical contexts as useful and facilitative for patient care.

Importantly, many students’ comments revealed the emergence of skills in “modularizing” components of the H&P 360—using only those most relevant to a particular clinical context without feeling compelled to cover every domain.

Third, students need to *manage the emergent information through further inquiry or redirection.* As reflected in some student comments, medical students can feel compelled to fully elucidate or address the complex behavioral or social drivers of patients’ health once identified. They also recognized that some behavioral or social needs uncovered during this process are important but do not require immediate action. Students should then redirect the interview to address matters of immediate concern (eg, potentially serious symptoms or a plan for initial hospital treatment), while simultaneously developing a plan to address longer-term issues. This process of identifying, prioritizing, and guiding the interview to optimize both disease-specific and contextual information has been demonstrated in the area of diagnostic reasoning, where a clinician listens and generates hypotheses, gathers data to test these hypotheses, and, depending on the results, offers treatment or pursues further diagnostic action [31]. We suggest that the domain-based framework of the H&P 360 facilitates the application of advanced interview skills not only to diagnostic assessment but also to management and care planning that better account for patients’ psychosocial and environmental realities.

Finally, the *new information elicited with the H&P 360 must be applied to clinical management planning.* Our data suggest that, while learners generally found that information from the H&P 360 enhanced care planning, ratings in this area were lower than for other measures and lower than those observed in previous studies [3,17], particularly among early learners. We believe these findings highlight the complexity of incorporating social and behavioral information into care planning—for example, in discharging a patient facing homelessness or supporting medication adherence limited by insurance, income, transportation, or behavioral factors. By bringing these “background” issues to the forefront early in training, students can develop skills to mobilize interprofessional teams and utilize local resources as part of routine patient care. We also anticipate that by directly addressing SDOH—rooted in racial, ethnic, and socioeconomic inequities—learners will be better equipped to recognize and manage systemic and personal implicit biases that negatively impact care.

Although not emphasized in the student-oriented results presented here, our study suggested that faculty play an important role in promoting the effective use of the H&P 360. Faculty development and feedback methods varied across participating sites, ranging from interactive seminars to entirely email-based communication, and faculty “buy-in” likely varied both within and across sites. At all sites, faculty were provided with information on the purpose, content, and suggested best practices for using the H&P 360. Anecdotally, however, learners reported little awareness or receptivity among teaching faculty toward using the H&P 360 domain framework in teaching and clinical management—except at 1 site where in-person faculty development was conducted before the COVID-19 pandemic. As professional development is influenced by cues from influential social sources, as well as practice resources and norms [22], effective application of the H&P 360 will likely require its incorporation into local teaching and clinical practices. Faculty development and the use of the 7-domain framework in teaching and clinical practice represent important areas for future investigation.

### Limitations

Our study was limited by small sample sizes, which prevented more rigorous statistical analyses of the Likert-type scale data and further qualitative analysis. However, both in this work and in previous studies, student responses to the H&P 360 have been primarily positive. Additionally, it is important to acknowledge that some students experienced difficulties implementing the H&P 360 during virtual interactions and in specific clinical encounters. More implementation training on how to utilize the H&P 360 in different scenarios might be helpful for students. The survey also had a low response rate at some institutions, which may be attributable to several factors. The survey was optional and not required at all 3 sites. In some cases, faculty were unable to administer the survey immediately after course completion due to time constraints.

Lastly, this project took place at the beginning of the pandemic, which introduced many competing priorities and adjustments to the overall learning environment. The overall impact of response rates on the results is difficult to estimate, as rates varied by institution and were likely influenced by additional local factors. Information on factors that could promote or limit the effective application of the H&P 360 was not explored beyond the data collected from the student surveys. For example, curricular content encountered by students before the H&P 360, as well as organizational culture, could influence its application at each institution. Exploration of these factors was outside the scope of this study. Survey data were limited by too few observations in relevant substrata (eg, inpatient vs outpatient; longitudinal vs short-term; virtual vs face-to-face; and institutional vs community-based clinical settings) to permit meaningful subgroup analyses exploring additional variables that may impact the implementation of the H&P 360 in different settings.

### Conclusions

The H&P 360 provides an enhanced template for data gathering that includes general prompts addressing key dimensions of human health not captured by the traditional H&P, such as patients’ values, priorities, and goals. Our findings support the usability of the H&P 360 as a more comprehensive approach for medical students to gather patient information. Among early learners, it may be best to include a few specific illustrative items under each domain to familiarize students with the domains without requiring higher-order clinical knowledge or skills. Among later learners, the now-familiar domains can be used to promote more complete data gathering and to develop skills in integrating patients’ goals, psychosocial and behavioral factors, and interprofessional teams into care planning. The H&P 360 may be particularly useful for making health inequities and their root causes more visible in routine clinical encounters, while guiding management planning to address them. Future work should measure its effects on patient care and outcomes.

Relevant topics for future investigation related to the H&P 360 include influences on students’ use of the H&P 360 at different developmental stages; its use to identify and address SDOH; and methods and outcomes of faculty development to promote routine incorporation of domain-based thinking into clinical teaching and practice. To facilitate further investigation and implementation of the H&P 360 among medical schools, a set of tools and resources is available on the AMA website or authors may be contacted directly for further information.

## Appendix

Multimedia Appendix 1 The H&P 360 template.

Multimedia Appendix 2 Student comments.

Multimedia Appendix 3 The CHERRIES (Checklist for Reporting Results of Internet E-Surveys) checklist.

Multimedia Appendix 4 Interview guide to support the H&P 360.

## Abbreviations

AMA: American Medical Association
CDC: Centers for Disease Control and Prevention
FIU: Florida International University
H&P: history and physical
LGBTQ+: lesbian, gay, bisexual, transgender, queer or questioning, and other diverse sexual orientations and gender identities
SDOH: social determinants or social drivers of health
UC: University of Chicago
UM: University of Michigan
WHO: World Health Organization
