# Supplementary material for: Implementing the H&P 360 in Three Medical Institutions: Usability Study
Source: JMIR Med Educ. 2025 Jun 5;11:e66221. doi: 10.2196/66221 (PMC12179563; doi:10.2196/66221)
Supplement: Multimedia Appendix 1 [file mededu_v11i1e66221_app1.docx]

**Reasons for visit:**


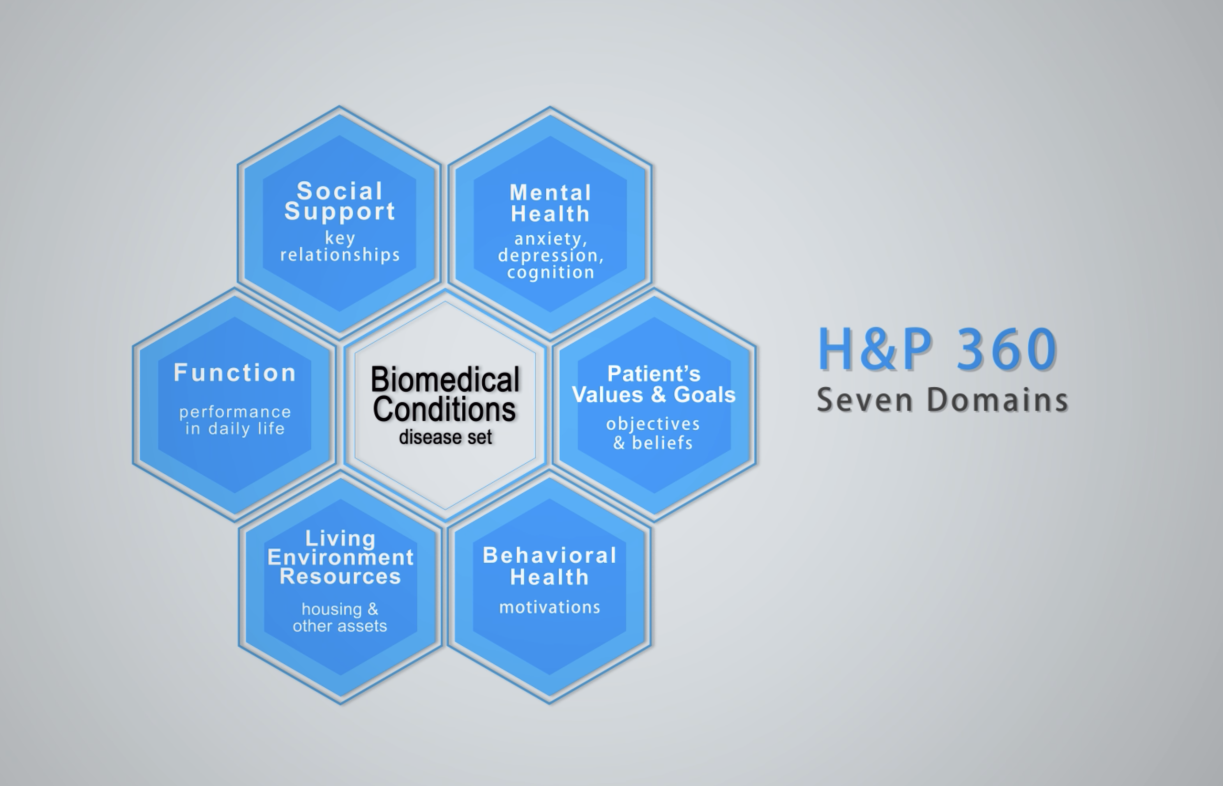


**History of present illness** (Is this a new patient? If yes, complete full history. If not, document pertinent changes)

1. **Biomedical problems/concerns:**
2. **Patient perception of health, priorities, and goals** (This domain encompasses: patient understanding/insight of illness/health, patient self-assessed level of control, patient-identified strengths and barriers):
3. **Mental health** (This domain encompasses: mood, thought patterns, diagnosed or undiagnosed psychiatric disorders, as well as pertinent social issues):

**Social history**

1. **Behavioral health** (This domain encompasses: health behaviors, medication management/adherence, nutritional behaviors, physical activity habits, personality disorders, substance use):
2. **Social support** (This domain encompasses: primary relationships, social support, caregiver availability, abuse/violence, community relationships):
3. **Living environment resources** (This domain encompasses: food security, housing stability, financial resources, transportation):
4. **Functional status** (This domain encompasses: affect, social and occupational functioning, satisfaction with life, activities of daily living):

**Past medical history:**

**Health maintenance (preventative care):**

**Past surgical history:**

**Family history:**

**Medications:**

**Allergies:**

**Review of systems:**

Constitutional:

Ear, nose, mouth, & throat:

Cardiovascular:

Respiratory:

Gastrointestinal:

Genitourinary:

Musculoskeletal:

Integumentary:

Neurological:

Psychiatric:

Endocrine:

Hematologic/lymphatic:

Allergic/immunologic:

**Physical exam:**

**Data:**

**Assessment/Plan** (problem-focused, with each problem receiving discussion of assessment and plan)

**Problem assessment** (problems can include issues that are primarily biomedical or issues that are psychosocial)

- Shared assessment of level of control
- Trajectory of condition (this includes relevant history, current condition status, condition outlook)
- Shared goal
- Psychosocial influences(including patient strengths and barriers)

**Plan**

- Team actions
  - Clinical (eg, specialist referrals, inter-professional team roles)
  - External (eg, community resources)
- Patient/family (eg, self-management)
- Therapy/monitoring
- Disposition/follow-up

Problem #1

Assessment:

Plan:

Problem #2

Assessment:

Plan:

Problem #3

Assessment:

Plan:

Problem #4

Assessment:

Plan:
