# Supplementary material for: Implementing the H&P 360 in Three Medical Institutions: Usability Study
Source: JMIR Med Educ. 2025 Jun 5;11:e66221. doi: 10.2196/66221 (PMC12179563; doi:10.2196/66221)
Supplement: Multimedia Appendix 2 [file mededu_v11i1e66221_app2.docx]

**Appendix 1: Student Comments**

| Required practice | Post clerkship student | *“I found it challenging to be thorough and cover all of the parts of the H&P360 in a quick clinic visit so I would usually pick one or two which seemed to be most relevant and go from there.”* |
| --- | --- | --- |
|  | Post clerkship student | *One challenge I found was that this style of interview brings up a lot of heavy and personal information and it can be difficult to hear things and then have to move on. This practice of compassionate listening but gentle redirection became easier with practice but was still challenging.”* |
|  | Post clerkship student | *“The full HP360 was long and unrealistic to include in every patient encounter. I never asked every component of the HP360, but I tried to incorporate some points into every encounter.”* |
| Clinical impact | Post clerkship student | *“[The H&P 360]...provided a framework to cover the basic categories of social well-being. Also, easy to use and see the impact of tailored interventions based on social situations, which certainly improves care.”* |
|  | Post clerkship student | *“I think [the H&P 360]...provides a framework for getting to know the patient better and helps to connect with them on a stronger and more genuine level.”* |
| Better clinician | Post clerkship student | *“[The H&P 360 was helpful in]... [r]eturning the humanity to medicine: patients are people first - Helping to understand some of the barriers to health and disease prevention that might not otherwise be apparent.”* |
|  | Clerkship student | *“Everyone should do it [use the H&P360], and think about these frameworks when talking to patients - these frameworks help us approach patient interactions in a trauma-informed manner.”* |
|  | Clerkship student | *“Facilitated stronger patient provider relationship, helped create comprehensive problem list”* |
| Benefits | Clerkship student | *“HP360 facilitated a deeper personal connection with the patient. I very much believe that it can enhance the Doctor-patient relationship.”* |
|  | Post clerkship student | *“1) The expanded social history portions were useful, and it was good to have a reminder to spend more time on the social history. 2) Asking about resources provided useful information for discharge planning.”* |
|  | Clerkship student | *“1. Increased rapport and trust with patients. 2. Identified high risk situations (e.g. elderly patient living alone) I otherwise might have missed.”* |
