# Supplementary material for: Implementing the H&P 360 in Three Medical Institutions: Usability Study
Source: JMIR Med Educ. 2025 Jun 5;11:e66221. doi: 10.2196/66221 (PMC12179563; doi:10.2196/66221)
Supplement: Multimedia Appendix 3 [file mededu_v11i1e66221_app3.pdf]

Cherries Checklist for H&P 360
Manuscript 2_25_2025

**Checklist for Reporting Results of Internet E-Surveys (CHERRIES)**			
**Item Category**	**Checklist Item**	**Explanation**	**Reporting Results**
Design	Describe survey design	Describe target population, sample frame. Is the sample a convenience sample? (In "open" surveys this is most likely.)	Convenience sample
Institutional Review Board approval and informed consent process	IRB approval	Mention whether the study has been approved by an IRB.	Approval was sought from the affiliated universities ethics committees.<br><br>UC: This study received exemption from IRB approval given minimal risk<br><br>UM: This study received exemption from IRB approval given minimal risk.<br><br>FIU: This study received exemption from IRB approval, given the minimal risk.<br>The IRB also granted approval for FIU to share de-identified data with the other AMA investigators.
	Informed consent	Describe the informed consent process. Where were the participants told the length of time of the survey, which data were stored and where and for how long, who the investigator was, and the purpose of the study?	Brent Williams was the principal investigator (PI) for the University of Michigan. Joyce Tang was the PI for the University of Chicago. Julia Bisschops was the PI for Florida International University. Deidentified survey data was stored on local servers and access was restricted to the approved project team members.<br><br>Due to the IRB exempt status, informed consent was not needed or explicitly solicited. Students completed surveys as a part of routine evaluation of teaching curricula.

	Data protection	If any personal information was collected or stored, describe what mechanisms were used to protect unauthorized access.	The survey platform (Qualtrics) uses encryption processes with restricted access authorization to protect all data collected. Only the research team can access the password-protected platform.
Development and pre-testing	Development and testing	State how the survey was developed, including whether the usability and technical functionality of the electronic questionnaire had been tested before fielding the questionnaire.	The survey was developed collaboratively across all three sites using a pre-determined blueprint. Individual items were reviewed for sensibility. At UC and UM, a small number of residents and students not involved in the study reviewed for sensibility. At FIU, items were reviewed for applicability by the research team. Electronic administration was through student feedback mechanisms already in place at each school. At FIU, electronic administration was through Qualtrics surveys sent to all relevant students and faculty via email.

Recruitment process and description of the sample having access to the questionnaire	Open survey versus closed survey	An “open survey” is a survey open for each visitor of a site, while a closed survey is only open to a sample which the investigator knows (password-protected survey).	It was an open survey but was only shared with students that participated in this study.
	Contact mode	Indicate whether or not the initial contact with the potential participants was made on the Internet. (Investigators may also send out questionnaires by mail and allow for Web-based data entry.)	UC: Initial contact with the potential participants was done via email.<br><br>UM: Initial contact with students was at an in-person seminar.<br><br>FIU: Initial contact with students was via online asynchronous training. Initial contact with faculty was via in-person training, followed by an always available online asynchronous module.
	Advertising the survey	How/where was the survey announced or advertised? Some examples are offline media (newspapers), or online (mailing lists – If yes, which ones?) or banner ads (Where were these banner ads posted and what did they look like?). It is important to know the wording of the announcement as it will heavily influence who chooses to participate. Ideally the survey announcement should be published as an appendix.	The survey was announced during classroom instruction. Students were informed of the survey, its timing, and how to access it when prompted via email.<br><br>FIU: The survey was announced during classroom instruction, which was virtual at the time, due to the pandemic. Students were informed of the survey, its timing, and how to access it when prompted via email.
Survey administration	Web/E-mail	State the type of e-survey (e.g., one posted on a Web site, or one sent out through e-mail). If it is an e-mail survey, were the responses entered manually into a database, or was there an	The survey was sent out via email and responses were collected via Qualtrics.

		automatic method for capturing responses?	
	Context	Describe the Web site (for mailing list/newsgroup) in which the survey was posted. What is the Web site about, who is visiting it, what are visitors normally looking for? Discuss to what degree the content of the Web site could pre-select the sample or influence the results. For example, a survey about vaccination on a anti-immunization Web site will have different results from a Web survey conducted on a government Web site	N/A
	Mandatory/ voluntary	Was it a mandatory survey to be filled in by every visitor who wanted to enter the Web site, or was it a voluntary survey?	Voluntary
	Incentives	Were any incentives offered (e.g., monetary, prizes, or non-monetary incentives such as an offer to provide the survey results)?	No incentives were offered.
	Time/Date	In what timeframe were the data collected?	UC: Data was collected between 5/2020-6/2021<br><br>UM: Data were collected between 2/2020 and 2/2021<br><br>FIU: Data were collected between 01/2021 and 04/2021.
	Randomization of items or questionnaires	To prevent biases items can be randomized or alternated.	N/A

	Adaptive questioning	Use adaptive questioning (certain items, or only conditionally displayed based on responses to other items) to reduce number and complexity of the questions.	N/A
	Number of Items	What was the number of questionnaire items per page? The number of items is an important factor for the completion rate.	The number of questionnaire items per page ranged from one to seven.
	Number of screens (pages)	Over how many pages was the questionnaire distributed? The number of items is an important factor for the completion rate.	The questionnaire was distributed over two pages.
	Completeness check	It is technically possible to do consistency or completeness checks before the questionnaire is submitted. Was this done, and if "yes", how (usually JAVAScript)? An alternative is to check for completeness after the questionnaire has been submitted (and highlight mandatory items). If this has been done, it should be reported. All items should provide a non-response option such as "not applicable" or "rather not say", and selection of one response option should be enforced.	A completeness check was implemented via Qualtrics. Participants were prompted to either check non-applicable or answer the question.
	Review step	State whether respondents were able to review and change their answers (e.g., through a Back button or a Review step which displays a summary of the responses and asks the respondents if they are correct).	Yes, respondents were able to review and change their answers prior to submitting the survey.

Response Rates	Unique site visitor	If you provide view rates or participation rates, you need to define how you determined a unique visitor. There are different techniques available, based on IP addresses or cookies or both.	N/A
	View rate (Ratio of unique survey visitors/unique site visitors)	Requires counting unique visitors to the first page of the survey, divided by the number of unique site visitors (not page views!). It is not unusual to have view rates of less than 0.1 % if the survey is voluntary.	N/A
	Participation rate (Ratio of unique visitors who agreed to participate/unique first survey page visitors)	Count the unique number of people who filled in the first survey page (or agreed to participate, for example by checking a checkbox), divided by visitors who visit the first page of the survey (or the informed consents page, if present). This can also be called "recruitment" rate.	UC: 31 students<br><br>UM: 100%. (All 13 students who visited the first page of the survey completed the first page of the survey).<br><br>FIU: 100% of those who visited the first page of the survey completed it.<br>17 students
	Completion rate (Ratio of users who finished the survey/users who agreed to participate)	The number of people submitting the last questionnaire page, divided by the number of people who agreed to participate (or submitted the first survey page). This is only relevant if there is a separate "informed consent" page or if the survey goes over several pages. This is a measure for attrition. Note that "completion" can involve leaving questionnaire items blank. This is not a measure for how completely questionnaires were filled in. (If you need a measure for this, use the word "completeness rate".)	Uc: 31 students<br><br>UM: 100% (All 13 students who agreed to participate completed the second page of the survey)<br><br>FIU: 100% of those who visited the survey completed the last page.<br>17 students

Preventing multiple entries from the same individual	Cookies used	Indicate whether cookies were used to assign a unique user identifier to each client computer. If so, mention the page on which the cookie was set and read, and how long the cookie was valid. Were duplicate entries avoided by preventing users access to the survey twice; or were duplicate database entries having the same user ID eliminated before analysis? In the latter case, which entries were kept for analysis (e.g., the first entry or the most recent)?	To our knowledge, cookies were not used.
	IP check	Indicate whether the IP address of the client computer was used to identify potential duplicate entries from the same user. If so, mention the period of time for which no two entries from the same IP address were allowed (e.g., 24 hours). Were duplicate entries avoided by preventing users with the same IP address access to the survey twice; or were duplicate database entries having the same IP address within a given period of time eliminated before analysis? If the latter, which entries were kept for analysis (e.g., the first entry or the most recent)?	To our knowledge, the IP address of the client computer was not identified.
	Log file analysis	Indicate whether other techniques to analyze the log file for identification of multiple entries were used. If so, please describe.	N/A

	Registration	In “closed” (non-open) surveys, users need to login first and it is easier to prevent duplicate entries from the same user. Describe how this was done. For example, was the survey never displayed a second time once the user had filled it in, or was the username stored together with the survey results and later eliminated? If the latter, which entries were kept for analysis (e.g., the first entry or the most recent)?	N/A
Analysis	Handling of incomplete questionnaires	Were only completed questionnaires analyzed? Were questionnaires which terminated early (where, for example, users did not go through all questionnaire pages) also analyzed?	Only completed surveys were analyzed.
	Questionnaires submitted with an atypical timestamp	Some investigators may measure the time people needed to fill in a questionnaire and exclude questionnaires that were submitted too soon. Specify the timeframe that was used as a cut-off point, and describe how this point was determined.	N/A
	Statistical correction	Indicate whether any methods such as weighting of items or propensity scores have been used to adjust for the non-representative sample; if so, please describe the methods.	No weighting was used.
