# Supplementary material for: Implementing the H&P 360 in Three Medical Institutions: Usability Study
Source: JMIR Med Educ. 2025 Jun 5;11:e66221. doi: 10.2196/66221 (PMC12179563; doi:10.2196/66221)
Supplement: Multimedia Appendix 4 [file mededu_v11i1e66221_app4.docx]

**Chronic Disease Prevention and Management History and Physical Interview Guide**

**Purpose**

Patients with chronic diseases often become their own primary caregiver and it is imperative for providers to assess their patient’s strengths and needs that may affect their ability to do so. Effective chronic disease prevention and management requires an interdisciplinary team to join together to help the patient build their capacity to self-manage their condition and address any barriers they may face. The chronic disease prevention and management history and physical tool aims to help providers perform an in-depth assessment of patient strengths and needs in order to co-create an individualized, comprehensive prevention and management plan with the patient and their interdisciplinary team.

**Learning objectives**

- Utilize appropriate tools (i.e., expanded social history, chronic disease history and physical) to obtain patient-centered values, goals, and socio-behavioral-economic factors that influence chronic disease screening, prevention, and management decisions
- Apply the information gathered to co-create a comprehensive chronic disease management plan with the patient.

**How to use this tool**

- Think of the social history as a way to get to know your patient and their individualized health and social situation and needs
- Depending on the visit type and the setting, not all questions may need to be asked
- Depending on the visit type and setting, the order of the questions may vary but we do encourage you to keep the social questions towards the beginning of the interview to allow you the opportunity to learn about the patient’s individualized needs and work them into your assessment and plan
- Pertinent psychosocial issues may be considered as their own diagnosis deserving of an appropriate plan to address them
- Open-ended questions can help you to elicit more information from your patient which can help you work with them to co-create an individualized plan of care

**Overview of expanded history of present illness (HPI) and social history domains**

**Reasons for visit** This is very similar to what has traditionally been called the chief complaint. This section is intended to record the patient’s key reasons for seeking care at this encounter. It could be a typical complaint, like “sore throat”, or it could be other reasons such as “follow-up of high blood pressure,” “health maintenance visit”, or “to discuss problems with a medication.” There can be more than one!

**Expanded HPI**

**Biomedical problems and concerns** In this section, we want you to ask about any biomedical problems or concerns your patient may be experiencing. The way you assess this will likely vary based on whether this is a chronic or acute issue. If it is an acute issue you can use OLDCART (onset, location, duration, characteristics, aggravating factors, relieving factors, treatment) to gather the information you need. If it is a chronic issue you may ask questions about their symptoms, how long they’ve had the condition, and their current and past treatments, etc. These types of questions can help you assess the trajectory of the issue.

**Patient perception of health** (This domain encompasses: patient understanding/insight of illness/health, patient self-assessed level of control, patient-identified strengths and barriers). In this section, we want you to learn about your patient’s perception of their health. Are there cultural beliefs or preferences they have related to their disease? How well do they think their disease is controlled? Do they understand their disease and what they need to do to manage it? What strengths and barriers do they identify as being a benefit or hindrance to their health?

**Patient priorities & goals** In this section, we want you to learn about what motivates your patient to try to stay as healthy as they can. What goals do they have for their life and/or their health? The priorities and goals that you document here should be revisited in your Assessment and Plan. You are encouraged to ask these questions early in your interview so you can think of how you can leverage these priorities to help the patient reach their goals and adhere to the plan you co-create.

**Psychosocial problems/concerns (**This domain encompasses: mood, thought patterns, diagnosed or undiagnosed psychiatric disorders, as well as pertinent social issues). In this section, we want you to identify any psychosocial barriers your patient may be encountering. For instance, do they have any undiagnosed psychiatric disorders or an impaired mood that may affect their ability to adhere to medical recommendation or self-manage their disease? Are there new pertinent social issues that may affect their ability to adhere or self-manage such as a recent job loss or a death in the family? Note: you are encouraged to document pertinent psychosocial issues early in your HPI, but it may not be appropriate or comfortable to ask these questions early in your interview, especially if it is your first encounter with a patient. Use your judgment on how and when to ask about these issues during your interview.

**Social history**

**Behavioral** (This domain encompasses: health behaviors, medication management/adherence, nutritional behaviors, physical activity habits, personality disorders, substance use). In this section, we want you to assess your patient’s health behaviors and identify if there are any improvements that need to be made. Does your patient take their medications as prescribed, do they follow a diet appropriate for their disease, how physically active are they, do they have any personality disorders that may impair their ability to appropriately manage their condition?

**Relationships** (This domain encompasses: primary relationships, social support, caregiver availability, abuse/violence, community relationships). In this section, we want you to assess what kind of a support system is available for your patient. Who helps them when they need help? Who encourages them to adhere to a healthy lifestyle? Are they experiencing any violence or abuse in their relationships?

**Resources** (This domain encompasses: food security, housing stability, financial resources, transportation). In this section, we want you to assess if there are any barriers that might be affecting your patient’s ability to manage their condition well. Does your patient need to prioritize putting food on the table or ensuring they have a roof over their head over paying for their medications? Do they have a way to get to the pharmacy or their appointments?

**Functional status** (This domain encompasses: affect, social and occupational functioning, satisfaction with life, activities of daily living). In this section, we want you to how well your patient is functioning in their day to day life. What is their affect? Are they effectively coping with their situation? Are they able to perform their activities of daily living independently or do they need help?

**Sample questions**

Some questions in the social history may be sensitive in nature and it is important to remain non-judgmental when asking them. To help with this, we have compiled a list of a few guiding questions you can use until you become more comfortable asking these types of questions.

**Behavioral**

- How many days per week do you get at least 30 minutes of exercise?
- What issues have you had taking your medication as prescribed?
- How many doses of medication have you missed in the past week?
- What issues have you had sticking to the healthy lifestyle recommendations given at your last visit?
- Do you ever use alcohol or drugs to deal with the stresses in life?

**Relationship**

- Who do you turn to when you feel the need for support?
- Are you afraid you might be hurt in your apartment building or house?
- Who do you rely on when you are unable to do something yourself?
- What community resources or programs do you use to improve or maintain your health?

**Resources**

- In the last 3 months, did you ever eat less than you felt you should because there wasn’t enough money for food?
- Do you have trouble affording foods that are part of a balanced diet?
- Are you worries that in the next 3 months you may not have stable housing?
- In the last month, have you slept outside, in a shelter, or in a place not meant for sleeping?
- How often in the past 12 months would you say you were worried or stressed about having enough money to pay your rent/mortgage?
- In the last 3 months has your utility company shut off your service for not being able to pay the bills?
- In the last 3 months, have you needed to see a doctor but could not because of cost?
- In the last 3 months have you ever had to go without medication or health care because you did not have a way to get to the pharmacy or doctor’s office?
- Are you concerned that you may lose your insurance coverage in the near future?
- Are you regularly able to get a friend or relative to take you to the pharmacy or to your doctor’s appointments?

**Functional status**

- Do you consistently feel overwhelmed by life’s stresses?
- How satisfied are you with your life?
- How often have you needed to ask for help doing daily activities (i.e. cooking, bathing, etc.)? Who do you ask for help when you need it?
- How would you rate your interactions with others?
  - Do you have close relationships?
  - Do you have difficult or complicated relationships?
- Are you working or in school?
  - In the past 12 months, how many times were you absent from school or work?
- How would you rate your ability to deal with life’s stresses?
